# Editorial: The future of psychology: Approaches to enhance therapeutic outcomes

**DOI:** 10.3389/fpsyg.2022.1116204

**Published:** 2022-12-20

**Authors:** Dawson Church, Oliver Baumann, Peta Stapleton

**Affiliations:** ^1^National Institute for Integrative Healthcare, Fulton, CA, United States; ^2^School of Psychology, Bond University, Gold Coast, QLD, Australia

**Keywords:** energy psychology, somatic interventions, psychotherapy, Emotional Freedom Techniques (EFT), fourth wave

A greater acceptance of mind-body approaches in psychology is emerging. Research indicates that supplementing established and evidence-based psychological techniques (e.g., behavioral, exposure and cognitive processing) with physiological or somatic interventions—such as acupressure, meditation, yoga and biofield therapies—enhances therapeutic outcomes.

Characterized as the “fourth wave” of psychology (Gallo, 2009), these therapies view psychological problems as interactions involving energy fields. This is the reason why they are collectively referred to as energy psychology. Energy fields have become essential tools in treatment and diagnosis, with devices such as EEGs, fMRIs, MEGs, PEMS, and TENS machines providing medicine with a new window of understanding into the functioning of brain and body. Among the techniques that produce changes in these energy fields are meditation, the stimulation of acupoints, neurofeedback, eye movements, imagery and intention.

By 2022, more than 400 papers on these therapies had been published in peer-reviewed journals, including meta-analyses, randomized controlled trials, and outcome studies. They demonstrate the efficacy of these approaches for conditions such as anxiety, depression, PTSD, with treatment effects often an order of magnitude greater than conventional therapies. Moreover, therapies in the counseling space focused on the relationships between the mind, body, brain, and behavior are now being recognized as effective treatments by various official bodies, including the US Veterans Administration, the UKs National Institute for Clinical Excellence, and the World Health Organization.

The studies in this Research Topic examine the evidence for the efficacy of interventions supplementing traditional approaches such as talk therapy.

In their Hypothesis and Theory article, Chan et al. propose a temporospatial neuroscientific model of the brain and self, which provides a detailed description of the temporal structure of transitive psychological processes that take place during psychotherapy and ultimately lead to healing. More specifically, the model provides guidance for when specific psychotherapeutic techniques are optimally employed to elicit neuroplastic changes in the brain, leading to neural activity changes associated with more adaptive, coherent, and energetic thought patterns and behavior.

Focusing on the psychological consequences of catastrophic events, Feinstein reviews the evidence for the effects of acupressure-based energy psychology techniques, on disorders such as PTSD, anxiety, and depression. Findings across more than 30 countries indicate energy psychology has immediate and long-lasting benefits and has led to the reduction or even complete elimination of disaster-related psycho-symptomatology. Feinstein's review further highlights evidence for the underpinning mechanisms of those treatment effects, with several imaging studies indicating that changes in brain activity accompany cognitive shifts.

The effects of another energy healing technique, Reiki, which involves a therapist placing their hands on or close to a client's body to activate self-healing processes, are evaluated in a systematic review by Zadro and Stapleton. The systematic search yielded 14 high-quality randomized controlled trials. These showed, compared to placebo, Reiki had substantial and significant effects on symptoms of depressed mood, anxiety, stress and burnout, without producing adverse effects.

Church, Stapleton, Gosatti et al. investigated the effects of EcoMeditation—a novel form of mediation that involves a sequence of active physical tasks to reduce mind wandering—on measures of psychological wellbeing as well as the experience of flow and transcendent states. Studies are increasingly measuring not only reductions in dysphoria, but the acquisition of elevated states such as happiness and transcendence. A pre-post comparison found both reductions in adverse psychological conditions and significant increases in flow and peak states. In a second study, Church, Yang et al. identified the beneficial effects of guided meditation on a range of objective markers (EEG, cortisol, and immunoglobin) that correlated with improvements in psychological symptoms related to PTSD, depression and somatic symptoms. They also proposed a new standard for evaluating EEG datasets, the ratio of delta to high frequency beta brain waves.

In a registered clinical trial, Krings et al. investigated a novel treatment approach for depression, combining Behavioral Activation Treatment (BATD) with an Attention Training Technique (ATT). Results showed positive effects on all aspects of depression symptomatology in the short-term (i.e., 2 weeks) but not longer-term (i.e., 3 months). Results further indicated that concurrent delivery of both treatments was more effective than sequential delivery. Booster sessions may potentially prolong the positive treatment effects.

Hauber and Boon provides an interesting contrast by not focusing on the therapeutic method but on how the nature of first-session therapeutic relationship has the potential to enhance psychotherapeutic outcomes. Using a sample of high-risk adolescents, the authors found that therapeutic relationship quality significantly positively affected treatment outcomes. High-quality first-session therapeutic relationships lead to a doubling of treatment effectiveness, while low-quality relationships rarely lead to positive outcomes.

Related to this work, Meier analyzed the causes of ceiling effects (positive-end response clusters) in measures of the therapeutic alliance (i.e., the quality of the relationship between the therapist and their client). The study analyzed self-report response patterns of drug-abuse patients drawn from an archival database. Replicating previous research they found ceiling effects on measures of the therapeutic working alliance. However, the analysis suggests that these effects are due to genuine reflections of commonly positive experiences of the therapist-patient relationship, even though social desirability influences cannot be entirely neglected.

Finally, Church, Stapleton, Vasudevan et al. provide a systematic review of the effects of Emotional Freedom Techniques (EFT), the most widely-used form of energy psychology, on psychological and physiological disorders. This study updates an earlier meta-analysis by Church (2013) and analyses 41 additional randomized controlled trials. Results confirm the earlier investigation and show EFT is efficacious for a range of psychological conditions (e.g., anxiety, depression, phobias, and posttraumatic stress disorder), as well as physiological disorders (e.g., pain, insomnia, and autoimmune conditions). It should be considered a first-line evidence-based primary care treatment.

Taken together, these nine studies provide an indication of the future direction of the field. They show that the newest generation of therapies, and especially energy psychology, are producing outcomes that can make a profound impact on public health. They demonstrate that natural drug-free methods and effective psychotherapy are able to effectively treat a wide range of disorders, often in brief time frames and without the side effects of pharmaceuticals.

## Author contributions

All authors listed have made a substantial, direct, and intellectual contribution to the work and approved it for publication.

## References

[B1] Church D. (2013). Clinical EFT as an evidence-based practice for the treatment of psychological and physiological conditions. Psychology 4, 645. 10.4236/psych.2013.48092 36438382 PMC9692186

[B2] Gallo F. P. (2009). Energy psychology in rehabilitation: Origins, clinical applications, and theory. Energy Psychology: Theory, Research, & Treatment 1, 57–72. 10.9769/EPJ.2009.1.1.FPG

